# Identification of two novel cytolysins from the hydrozoan *Olindias sambaquiensis* (Cnidaria)

**DOI:** 10.1186/1678-9199-20-10

**Published:** 2014-03-25

**Authors:** Vidal Haddad Junior, Fernando Zara, Sergio Marangoni, Daniela de Oliveira Toyama, Alex Jardelino Felizardo de Souza, Simone Cristina Buzzo de Oliveira, Marcos Hikari Toyama

**Affiliations:** 1 São Paulo Experimental Coast Campus, São Paulo State University (UNESP – Univ Estadual Paulista), São Vicente, São Paulo State, Brazil; 2 Botucatu Medical School, São Paulo State University (UNESP – Univ Estadual Paulista), Botucatu, São Paulo State, Brazil; 3 Department of Biochemistry, Institute of Biology, State University of Campinas (UNICAMP), Campinas, São Paulo State, Brazil; 4 Center of Biological and Health Sciences, Mackenzie Presbyterian University, São Paulo, São Paulo State, Brazil; 5 UNESP, Campus do Litoral Paulista, Unidade São Vicente, Praça Infante D. Henrique, s/n, São Vicente, SP, CEP 11330-900, Brasil

**Keywords:** *Olindias sambaquiensis*, Hemolytic, Myonecrosis, Cytotoxicity, Cytolysin, Cnidaria venom

## Abstract

**Background:**

Although the hydrozoan *Olindias sambaquiensis* is the most common jellyfish associated with human envenomation in southeastern and southern Brazil, information about the composition of its venom is rare. Thus, the present study aimed to analyze pharmacological aspects of *O. sambaquiensis* venom as well as clinical manifestations observed in affected patients. Crude protein extracts were prepared from the tentacles of animals; peptides and proteins were sequenced and submitted to circular dichroism spectroscopy. Creatine kinase, cytotoxicity and hemolytic activity were evaluated by specific methods.

**Results:**

We identified two novel cytolysins denominated oshem 1 and oshem 2 from the tentacles of this jellyfish. The cytolysins presented the amino acid sequences NEGKAKCGNTAGSKLTFKSADECTKTGQK (oshem 1) and NNSKAKCGDLAGWSKLTFKSADECTKTGQKS (oshem 2) with respective molecular masses of 3.013 kDa and 3.375 kDa. Circular dichroism revealed that oshem 1 has random coils and small α-helix conformation as main secondary structure whereas oshem 2 presents mainly random coils as its main secondary structure probably due to the presence of W (13) in oshem 2. The hemolysis levels induced by oshem 1 and oshem 2 using a peptide concentration of 0.2 mg/mL were, respectively, 51.7 ± 6.5% and 32.9 ± 8.7% (n = 12 and p ≤ 0.05). Oshem 1 and oshem 2 showed significant myonecrotic activity, evaluated by respective CK level measurements of 1890.4 ± 89 and 1212.5 ± 103 (n = 4 and p ≤ 0.05). In addition, myonecrosis was also evaluated by cell survival, which was measured at 72.4 ± 8.6% and 83.5 ± 6.7% (n = 12 and p ≤ 0.05), respectively. The structural analysis showed that both oshem 1 and oshem 2 should be classified as a small basic hemolytic peptide.

**Conclusion:**

The amino acid sequences of two peptides were highly similar while the primary amino acid sequence analysis revealed W (22th) as the most important mutation. Finally oshem 1 and oshem 2 are the first cytolytic peptides isolated from the *Olindias sambaquiensis* and should probably represent a novel class of cytolytic peptides.

## Background

**Figure 1:**
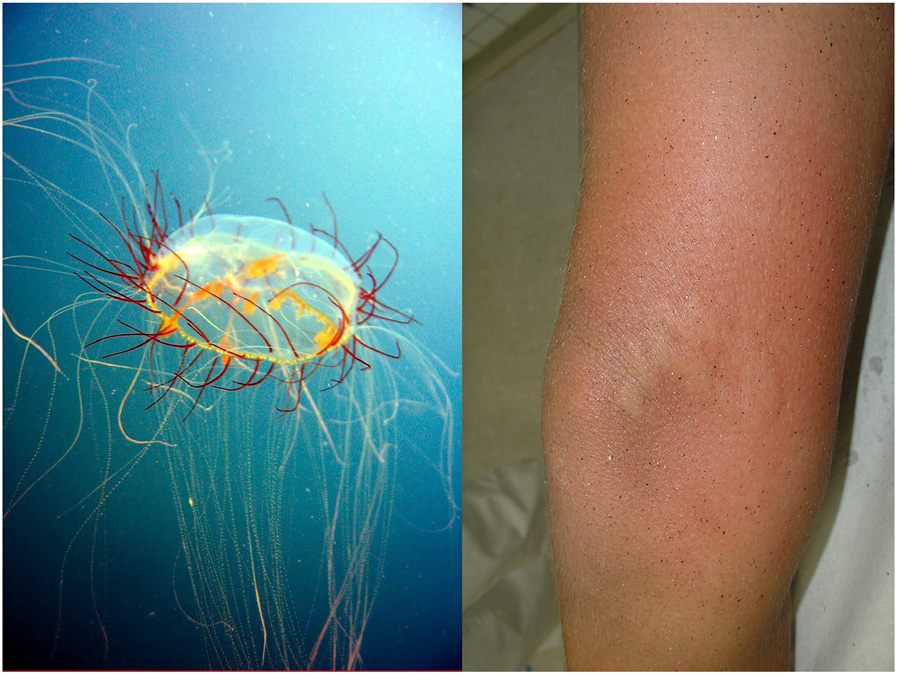
***Olindias sambaquiensis *and a typical lesion in a human.** Photos: Álvaro E Migotto and Vidal Haddad Junior.

The phylum Cnidaria is subdivided into five classes. Some species are important in human medicine since they can cause severe envenomation by the injection of venom through specialized cells. In Brazil, these organisms are included in the Hydrozoa, Scyphozoa and Cubozoa classes [1,2]. The class Hydrozoa has two species frequently associated with human envenomation: the Portuguese man-of-war (*Physalia physalis*) and *Olindias sambaquiensis* (Figure 1), the latter being related to the majority of accidents in the most populated regions of Brazil [1,2]. Like the other cnidarians, they present stinging cells equipped with small organelles known as nematocysts that contain small threads ejected along with the venom when stimulated mechanically or chemically [3]. Furthermore, nematocysts are found throughout the body of the cnidarians and not just in their tentacles [4-6].

*Olindias sambaquiensis* (“relojinho” in the Portuguese language), is a small hydrozoan jellyfish that provokes mild accidents on the southeastern and southern Brazilian coast where it is commonly found in winter and autumn [1,2,7]. Despite the medical relevance and abundance of this species in Brazil, there are not any studies characterizing its venom biochemically. The present work aimed to report the structural and biological characterization of hemolytic proteins from the nematocysts of *Olindias sambaquiensis*.

## Methods

The jellyfish were collected off the São Vicente beach (latitude 23º57'47" – South, longitude 46º23'31" – West) and their tentacles were then subjected to three freeze-thaw cycles. After the last freeze-thaw cycle, the solution was centrifuged at 20,000 g for 60 minutes at 4°C. Crude protein extracts were prepared from the tentacles removed from the jellyfish body using forceps and immediately immersed in an ice-cold 0.1% trifluoroacetic acid (TFA). The supernatant was recovered and filtered through a 0.45-μm filter, followed by a second ultrafiltration using a 0.22-μm filter.

The desired fraction was then lyophilized and the resulting peptides were subjected to hemolysis assay on freshly prepared human erythrocytes.

Protein sequencing was performed as previously described by Oliveira *et al.*[8]. Briefly, two milligrams of the purified protein was dissolved in 200 μL of a 6 mol/L guanidine chloride solution (Merck, Germany) containing 0.4 mol/L of Tris–HCl and 2 mmol/L of EDTA (pH 8.15). Nitrogen was blown over the top of the protein solution for 15 minutes, after which the protein solution was reduced with DTT (6 M, 200 μL) and subjected to a second incubation under a nitrogen atmosphere for 90 minutes. After this incubation time, 80 μL of iodoacetic acid was added to the solution (50 mM of cold iodoacetic and carboxymethylated ^14^C-iodoacetic acid), followed by a third incubation under a nitrogen atmosphere after which the reaction tube was sealed. To remove excess reagent and to purify the peptides, we used a preparative C5 reverse phase column.

Circular dichroism (CD) spectroscopy: the purified peptide was dissolved in 10 mM sodium phosphate pH 7.4 and the solution was adjusted to final protein concentration of 10 μM. After centrifugation at 4500 × *g* for five minutes, samples were transferred to a 1 mm path-length quartz cuvette. Circular dichroism spectra in the wavelength range 185–300 nm were acquired in-house on a J720 spectropolarimeter (Jasco Corp., Japan) using a bandwidth of 1 nm and a response time of 1 s. Data collection was performed at room temperature. With a scanning speed of 100 nm/min, a total of nine scans was accumulated for each sample and all the spectra were corrected by subtraction of buffer blanks.

The release of creatine kinase (CK) from damaged muscle cells was followed by use of the Kit 47-UV (Sigma-Aldrich Co., USA) to measure the enzyme activity in mouse plasma. For this determination five groups of mice (18–22 g) were injected in the right gastrocnemius muscle with 50 μL of 0.5 mg. mL^–1^ toxins (n = 4) while the control group received PBS. For the CK determination in plasma, the animals were lightly anesthetized with halothane immediately before and three hours after toxin or PBS injection for blood collection according to the guidelines for the care and use of laboratory animals [9].

Mice were bled from the tail three hours after injection and blood was collected into heparinized tubes, with one aliquot being used for CK determination. Plasma was separated by centrifugation and stored at 4°C for subsequent determination of CK activity. The procedure for the measurement of CK activity was conducted following the instructions of the kit (Kit 47-UV, Sigma Chemical Co., USA). Enzyme activity is reported as international units per liter (U/L), in which 1 U is the amount that catalyzes the transformation of 1 μmol of substrate at 25°C. The cytotoxicity assay was conducted according to Passero *et al*. [10] with toxins (or PBS as control) incubated with cells for 60 minutes. Cytotoxicity of the toxins from the *Olindias sambaquiensis* in J774 cells in culture was assayed using 3-(4,5-Dimethylthiazol-2-yl)-2,5-diphenyl tetrazolium bromide (MTT). Briefly, cells in 96-well plates (106 macrophage/well) in culture medium (RPMI 1640) for 48 hours at 35°C and 5% CO_2_ were incubated with various toxin concentrations ranging from 0.78 to 100.00 μg/well for 48 hours. The cells were then washed and incubated with MTT for four hours, followed by SDS with 10% HCl for the determination of formazan conversion, which was monitored by absorbance at 595 nm. This experiment was carried out in triplicate.

Hemolytic activity of the toxin was measured quantitatively in terms of attenuance on human red blood cells (RBC) at room temperature using a Spectramax Microtiter® plate reader (Molecular Devices, USA). Human blood, freshly collected with heparin, was centrifuged to remove the buffy coat, and the erythrocytes obtained were washed three times in 0.85% saline and stored at 4ºC. Toxins at desired concentrations were added in the first well to erythrocyte buffer (140 mM NaCl, 10 mM Tris–HCl, pH 7.4), and then serially diluted two-fold. RBCs (100 μL; D630 = 0.5) in erythrocyte buffer were added to the toxins, and hemolysis was monitored by measuring attenuance at 630 nm for 20 minutes at room temperature. The final volume was 200 μL per well. The hemolysis percentage was determined at the end of the assay using the following equation of Malovrh *et al*. [11]:

$$\mathrm{Hemolysis}\;\left(\%\right)\;\frac{\left(D\max-\mathrm{Dobs}\right)}{D\max-D\min}\times 100$$

in which Dobs was the measured attenuance in the well after 20 minutes and Dmax is the maximal attenuance by distilled water and Dmin is the minimal attenuance by buffer used in the test. For this assay we firstly determined the hemolytic CI50 values for oshem 1 and oshem 2, which were 324 μg and 198 μg, respectively.

The animal utilization was approved by the Committee for Ethics in Animal Experimentation of the Institute of Biology, UNICAMP, certificate number 1320–1. We utilized Swiss mice supplied by CEMIB, the Multidisciplinary Center for Biological Research of UNICAMP.

## Results and discussion

Accidents involving the hydrozoan *Olindias sambaquiensis* are common in South America and may provoke dermatitis, skin lesions, edema and pain. *Olindias sambaquiensis* poisoning is characterized as moderate to severe, which can lead to cardiovascular complications including cardiopulmonary arrest. Despite its clinical importance and the seriousness of symptoms observed, this is the first study of the biochemical characterization and isolation of toxic components from *Olindias sambaquiensis*.

**Figure 2:**
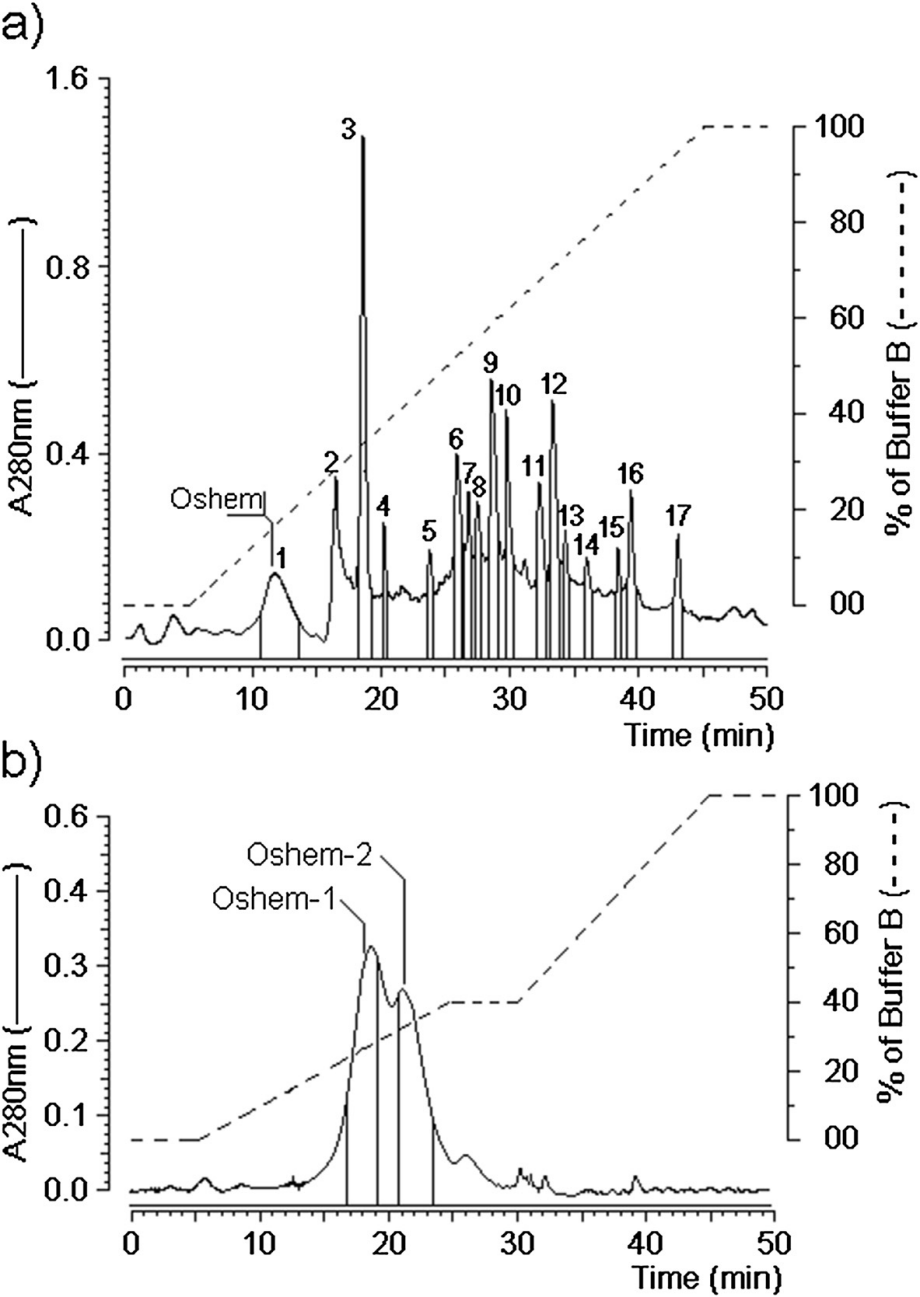
**Initial fractionation of protein extract obtained after addition of TFA on the *Olindia sambaquiensis *aqueous extract. (a)** The main hemolytic fraction was named oshem. **(b)** Re-purification of oshem by C18 reverse phase HPLC created two main fractions, oshem 1 and oshem 2.

The protocol used for this work essentially followed the methods used for *Bunodosoma caissarum*[12] and *Phyllorhiza punctata*[13]. The crude extract from *Olindias sambaquiensis* revealed the presence of at least 17 fractions, of which only one fraction, denominated oshem, showed a moderate hemolytic activity (Figure 2a), and was subjected to novel fractionation on C18 reverse chromatography using non-linear gradient of buffer B (66.6% of acetonitrile in 0.1% TFA) at a constant flow rate of 1.0 mL/min. Under this condition, we purified two isoforms from oshem that were designated oshem 1 and oshem 2 (Figure 2b).

Sea anemone toxins comprise mainly proteins and peptides that are cytolytic or neurotoxic with their potency varying according to the structure and action site, and are efficient at targeting different animals, such as insects, crustaceans and vertebrates. Summarily, the cnidarians venom includes 3.5 to 6.5 kDa voltage-gated sodium (NaV) channels toxins and 3–5 kDa voltage-gated potassium (KV) channel toxins and ~20 kDa pore-forming toxins [14]. In the case of pore-forming toxins, there are three other less frequent classes of toxins that can be classified as: group I, which consists of peptides from 5 to 8 kDa; group III with a molecular mass of 80 kDa; and the pore-forming toxins with phospholipase A2 activity and molecular mass around 20 to 40 kDa [15]. Toxins oshem 1 and oshem 2 contain hydrophilic low-molecular-weight peptides, and can be classified as group I toxins, which are peptides of 5 to 8 kDa, following the classification established by Anderluh and Macek [15].

**Figure 3:**
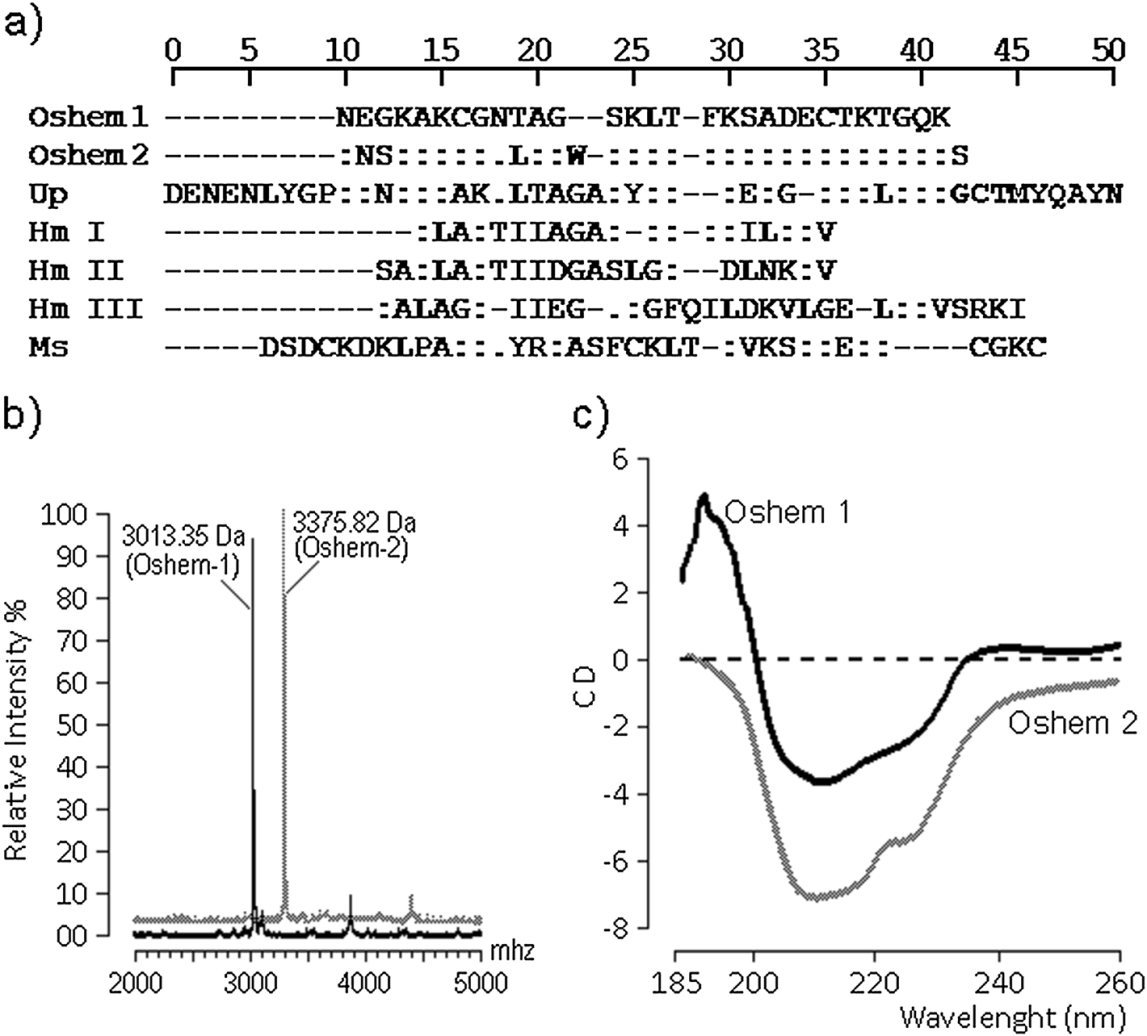
**Aminoacid sequences, mass spectrometry and CD spectra results of Oshem 1 and Oshem 2. (a)** Complete amino acid sequences of oshem 1 and oshem 2 are compared with other hemolytic and cytolysin isolates from *Urticina piscivora* (Up), *Heteractis magnifica* (magnificalysin I, magnificalysin II and magnificalysin III) and *Metridum senile* (Ms). **(b)** Mass spectrometry results of oshem 1 and oshem 2 with respective molecular masses of 3.013 kDa and 3.375 kDa. **(c)** CD spectra of oshem 1 and oshem 2 in the wavelength range of 185–300 nm were acquired in-house with a J720 spectropolarimeter (Jasco Corp., Japan) using a bandwidth of 1 nm and a response time of 1 s.

The N-terminal amino acid sequencing of oshem 1 and oshem 2 was determined after reduction. Both were subjected to automatic amino acid sequencing using a Procise® Protein Sequencer (Applied Biosystems, USA). The phenylthiohydantoin (PTH) derivatives of the amino acids were identified by an Applied Biosystems model 450 microgradient PTH analyzer. (Figure 3a) and a 50% amino acid identity with Up (*Urticina piscivora*). The molecular masses of oshem 1 and 2 were measured according to the description of Toyama *et al*. [16], and found to be, respectively, 3.013 kDa and 3.375 kDa (Figure 3b). The circular dichroism showed that oshem 1 presents an α-helix structure not found in oshem 2, which revealed mainly the presence of random coil structures (Figure 3c) while the most important shifts observed were L (10th) and W (13th), which should be involved in modification of the secondary structure between oshem 1 and oshem 2. This structural replacement from oshem 1 to oshem 2 induced a significant modification in the secondary structure of these toxins. The structural contents of oshem 1 comprise: percentage (%) by CD analysis of α-helix 33; β-sheet 48 and random coils 19; whereas the secondary structural contents of oshem 2 are: percentage (%) by CD analysis of α-helix 04; β-sheet 68 and random coils 28.

Data collection was performed at room temperature, with a scanning speed of 100 nm.min^−1^. Nine scans were accumulated for each sample whereas all spectra were corrected by subtraction of buffer blanks. The CD spectra are expressed as theta machine units in millidegrees.

**Figure 4:**
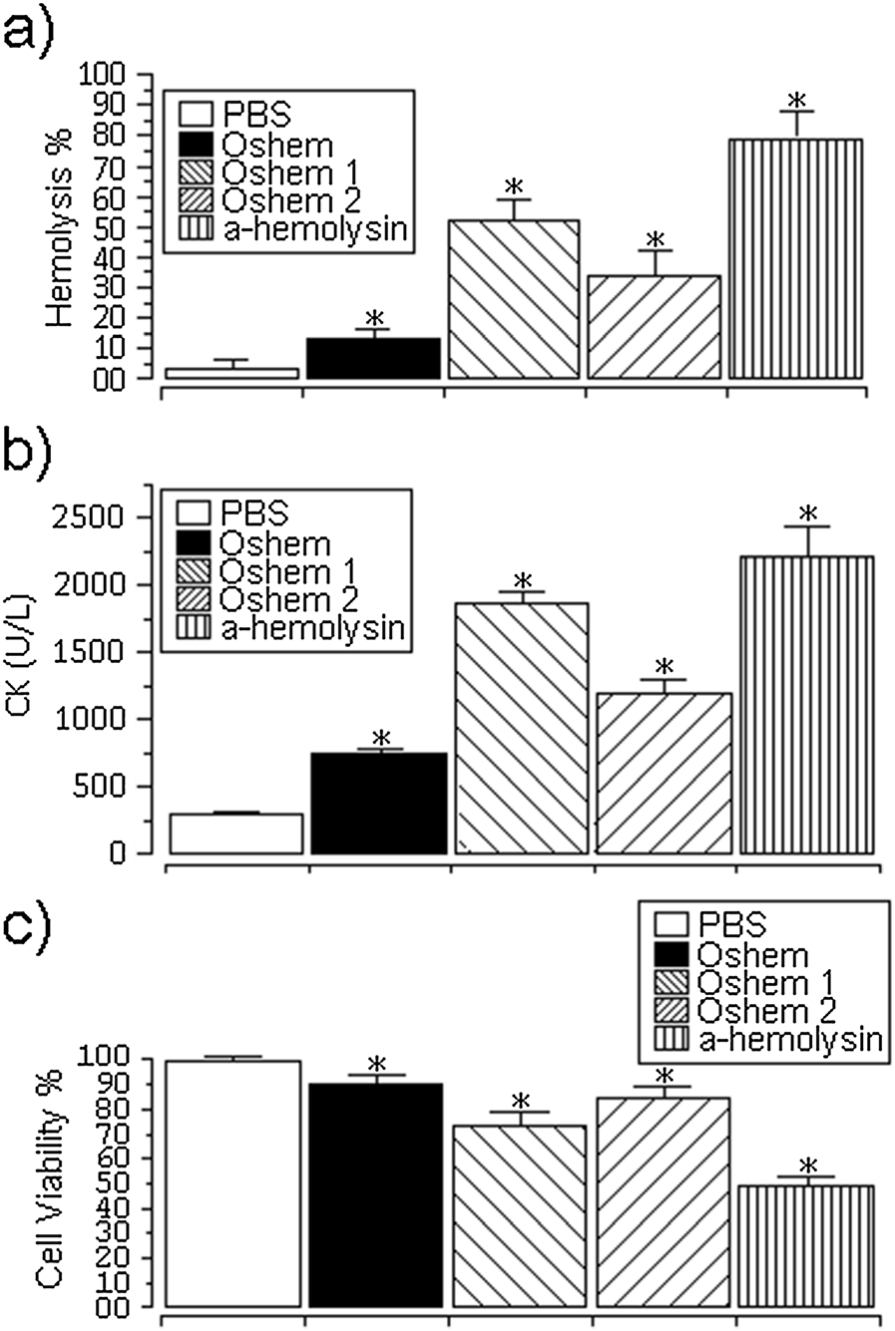
**Hemolytic effect of oshem, oshem1 and oshem 2 and alpha-hemolysin compared with alpha-hemolysin of *Staphylococcus aureus*. (a)** The hemolytic effect on the hRBC at 0.3 μmol of oshem, oshem-1 and oshem-2 and α-hemolysin (these results expressed as hemolysis/% were compared to hemolytic toxin isolated from the α-hemolysin from *Staphylococcus aureus*). Each point represents the mean ± SD of n = 12 experiments, with *p < 0.05. **(b)** The comparative effect of different proteins at the dose of 1 μM. **(c)** Macrophage cell viability expressed as cell viability percentage; data are presented as mean ± SEM (n = 12, p ≤ 0.05).

The hemolytic activity levels of oshem fraction before its fractionation in oshem 1 and oshem 2 was evaluated at 12.4 ± 3.4% (n = 12 and p ≤ 0.05), whereas the levels of oshem 1 and 2 were 51.7 ± 6.5% and 32.9 ± 8.7% (n = 12 and p ≤ 0.05), respectively, and the hemolytic potency of oshem 1 was two-fold less than that of α-hemolysin (Figure 4a). CK level after the injection of oshem was 759.50 ± 34 (n = 4, p ≤ 0.05), whereas treatment with oshem 1 or 2 increased CK levels by 1890.4 ± 89 and 1212.50 ± 103, respectively (n = 4, p ≤ 0.05) (Figure 4b). Cytotoxicity assay results were expressed as percentage of viable cells. Macrophage cell viability was moderately decreased in the presence of whole oshem fraction before its fractionation in oshem 1 and oshem 2 was 92.4 ± 3.4% (n = 12, p ≤ 0.05). For oshem 1 and 2 the values were 72.4 ± 8.6% and 83.5 ± 6.7% (n = 12 and p ≤ 0.05), respectively (Figure 4c). Cytolysins adopt a stable soluble structure, which undergoes a conformational change when brought into contact with a membrane, leading to an active, membrane-bound form that inserts spontaneously into the membrane [17-19]. The helix structure of oshem 1 appears crucial for exerting its full hemolytic activity, while the presence of W (22th) virtually destroyed its helix structure but did not abolish its hemolytic, myonecrotic or cytotoxic effect. The CD structure of oshem 2 showed the presence of random coil and beta-sheet structure, raising the possibility that beta sheet may make an important structural contribution to the biological and pharmacological activity of oshem 1 and 2.

The hemolysis percentage was calculated by the following formula:

$$\mathrm{Hemolysis}=\frac{\left(A540\;\mathrm{in}\;\mathrm{the}\;\mathrm{peptide}\;\mathrm{solution}–A540\;\mathrm{in}\;\mathrm{PBS}\right)}{\left(A540\;\mathrm{in}\;1\%\;\mathrm{Triton}-X100–A540\;\mathrm{in}\;\mathrm{PBS}\right)}\times 100\%$$

Figure 4 shows the measurement of CK levels after three hours of toxin injection. The analysis of the figure revealed that oshem 1 and oshem 2 induced a significant increasing of CK levels in relation to oshem purified from the first chromatography. But this myonecrotic effect was lower if compared to α-hemolysin from *Staphylococcus aureus*.

## Conclusions

*Olindias sambaquiensis* Muller, 1861 (Olindiidae) is a hydromedusae common in southern and southeastern Brazilian coastal regions and Atlantic coast of Argentina and Uruguay [7]. The accident caused by this jellyfish has been described as presenting mild pain, round plaques and no systemic symptoms [1,2,20,21]. Our results suggest that the oshem fractions exert significant pharmacological and toxic effects that can play an important role in the clinical manifestation induced by *O. sambaquiensis.*

### Ethics committee approval

The present study was approved by the Committee for Ethics in Animal Experimentation on the São Paulo Experimental Coast Campus of UNESP. The animal utilization was approved by the Committee for Ethics in Animal Experimentation of the Institute of Biology, UNICAMP, certificate number 1320–1. The utilized Swiss mice were supplied by CEMIB, the Multidisciplinary Center for Biological Research of UNICAMP.

## Competing interests

The authors declare that there are no competing interests.

## Authors’ contributions

TLP and VHJ were responsible for the clinical and experimental study of the venom; FJZ, SM, DOT, AFS, VCGS and SCBO carried out the immunoassays and participated in the sequence alignments; MHT was the senior author of the manuscript. All authors read and approved the final manuscript.
